# Microdosing psychedelics in the treatment of ADHD and comorbid disorders

**DOI:** 10.1192/j.eurpsy.2024.44

**Published:** 2024-08-27

**Authors:** K. P. Kuypers

**Affiliations:** Neuropsychology and Psychopharmacology, Maastricht University, Maastricht, Netherlands

## Abstract

**Abstract:**

Microdosing psychedelics has garnered considerable attention within both nonprofessional circles and the scientific community in recent years. This method involves taking small, non-hallucinogenic doses of substances like LSD or psilocybin over weeks or months, purportedly to enhance specific behaviors, emotions, or address psychiatric conditions.

Exploring these assertions is crucial given the potential therapeutic value of microdosing, especially in conditions that respond positively to full psychedelic doses, such as depression. The full psychedelic experience might not always be suitable due to various factors like age, capacity to consent or comprehend the experience (e.g., dementia), or individual personality traits that might hinder surrendering to the experience. Microdosing could potentially serve as a maintenance therapy post-full dose administration, aiding specific psychological or biological processes during therapy or therapeutic exercises.

Recent studies in healthy individuals highlight that small psychedelic doses have nuanced effects on pain perception, mood, neuroplasticity, sleep duration, brain connectivity, and default mode network synchronicity. However, some parameters show null effects after both single and repeated administration.

Our survey research uncovered that individuals with ADHD reported symptom relief through microdosing, deeming it more effective than their conventional treatments. Subsequently, we conducted a naturalistic study following individuals with ADHD across a 4-week microdosing period. Our findings indicated a reduction in symptoms over time, an increase in trait mindfulness, and a decrease in neuroticism compared to baseline. While these results are intriguing, they necessitate validation in a clinical trial. We have recently concluded such a trial and are currently analyzing the data to further explore these effects.

**Disclosure of Interest:**

K. Kuypers Grant / Research support from: The author is a principal investigator on a research project that is sponsored by Mindmed, a company that is developing psychedelic medicines.

